# Appropriateness of CT Pulmonary Angiography Requests at Croydon University Hospital: An Audit

**DOI:** 10.7759/cureus.99348

**Published:** 2025-12-16

**Authors:** Subodh Khadka, Osman Haji

**Affiliations:** 1 Endocrinology and Diabetes, Croydon Health Services NHS Trust, London, GBR; 2 General Medicine, NHS England, London, GBR

**Keywords:** ct angiography radiation exposure, ct pulmonary angiogram (ctpa), d-dimer levels, pulmonary embolism (pe), wells pe score

## Abstract

Background

CT pulmonary angiography (CTPA) is widely used in the evaluation of patients with suspected pulmonary embolism (PE); however, inappropriate requests can expose patients to unnecessary radiation and iodinated contrast, delay imaging for those with confirmed PE, and increase demand on radiology services. National guidance (National Institute for Health and Care Excellence (NICE) NG158) recommends applying the two-level Wells score and D-dimer testing to determine pretest probability before requesting CTPA. This two-cycle audit evaluated adherence to these standards at Croydon University Hospital and examined whether documentation and diagnostic pathway compliance improved over time.

Methods

A retrospective two-cycle audit was conducted. Cycle 1 reviewed all CTPAs requested for suspected PE between October 1 and 31, 2024. Cycle 2 repeated the audit in September 2025 using the same methodology and comparison standards. Data were extracted from the electronic health record and radiology system, including Wells score documentation, D-dimer testing, chest X-ray (CXR) findings, anticoagulation status, and CTPA outcomes. Audit standards were based on NICE NG158 and Royal College of Radiologists expectations for CTPA diagnostic yield (15-35%). Following Cycle 1, the Trust introduced mandatory Wells score and D-dimer fields in the Cerner CTPA request form and increased educational messaging regarding appropriate imaging.

Results

Cycle 1 included 58 patients, with 11 positive CTPAs (diagnostic yield, 19%). Wells score documentation was low, with only four documented cases (6.9%), and D-dimer testing was recorded in 45 requests (77%). In Cycle 2, 96 patients were included, with 16 positive CTPAs (diagnostic yield, 16.7%). Documentation improved markedly: Wells score recording increased to 84 cases (87.5%), and D-dimer documentation was present in 86 cases (89.6%). Among Cycle 2 patients, PE was detected in 14 of 61 patients (23%) with Wells scores greater than 4 and in two of 23 patients (8.7%) with Wells scores of 4 or less. Alternative diagnoses were identified on CXR in 25 cases (26%). Patterns of anticoagulation were similar between cycles.

Conclusions

Across two audit cycles, CTPA diagnostic yield remained stable and within the nationally expected range. However, documentation of the Wells score and D-dimer improved substantially by September 2025, reflecting better adherence to the recommended diagnostic pathway for suspected PE. These findings demonstrate the effectiveness of system-level changes, including mandatory request-form fields and improved clinician awareness. Continued monitoring is recommended to ensure sustained compliance and to support appropriate imaging utilization.

## Introduction

Pulmonary embolism (PE) is a potentially life-threatening condition resulting from obstruction of the pulmonary arteries, most commonly by thrombi originating in the deep veins of the lower limbs [1]. Prompt and accurate diagnosis is essential, as delayed or missed PE is associated with significant morbidity and mortality [2], whereas unnecessary investigations may expose patients to avoidable harm. CT pulmonary angiography (CTPA) is the gold standard investigation for the diagnosis of acute PE, offering high sensitivity and specificity, rapid acquisition, and widespread availability [3].

However, the convenience of CTPA has led to its overuse in many healthcare settings [4]. Inappropriate requests for imaging not only increase patient exposure to ionizing radiation and iodinated contrast but also impose considerable strain on radiology services and healthcare budgets [5]. Furthermore, excessive CTPA utilization has been shown to reduce diagnostic yield and increase the detection of clinically insignificant emboli, potentially leading to overtreatment [6]. In the United Kingdom, national guidance, including the National Institute for Health and Care Excellence (NICE) guideline NG158, recommends a structured, stepwise diagnostic approach incorporating pretest probability assessment using the two-level Wells score and D-dimer testing to determine the need for CTPA [7]. When this pathway is followed appropriately, the expected rate of positive CTPA examinations ranges between 15% and 35%, as cited by the Royal College of Radiologists (RCR) [7].

At Croydon University Hospital, a district general hospital in south London with over 500 beds and 41,000 admissions annually [8], anecdotal observation within the radiology and respiratory departments suggested frequent CTPA requests for patients with low clinical probability or without documented Wells scores or D-dimer results. In this context, an audit was initiated to assess local practice against national standards and determine whether CTPA was being used appropriately for suspected PE. The audit aimed to quantify the diagnostic yield of CTPA, evaluate adherence to guideline-recommended use of Wells scoring and D-dimer testing, and identify opportunities for improvement.

## Materials and methods

This project was conducted as a two-cycle retrospective clinical audit at Croydon University Hospital, a large district general hospital in south London. The audit aimed to assess adherence to national diagnostic standards for investigating suspected PE and to evaluate whether documentation and pathway compliance improved following local quality-improvement measures. The first cycle reviewed all CTPAs requested from October 1 to 31, 2024, and the second cycle from September 1 to 30, 2025, using the same methodology. The audit was registered with the Trust Clinical Audit Department (Audit ID 2025/117). Audit standards were derived from NICE NG158, which recommends that patients with suspected PE undergo assessment of pretest probability using the two-level Wells score. Patients classified as “PE likely” (score >4) should proceed directly to CTPA, whereas those categorized as “PE unlikely” (score ≤4) should undergo D-dimer testing, with imaging reserved for those with a positive result. The RCR states that, when such pathways are followed, between 15% and 35% of CTPAs should confirm PE. These standards were used as benchmarks for both cycles.

All consecutive patients undergoing CTPA for suspected PE during each audit period were included, with no exclusion criteria. Scans requested for alternative indications, such as suspected aortic pathology, were excluded. Data were extracted retrospectively from the electronic health record (Cerner) and the radiology information system. Information collected included Wells score documentation, D-dimer testing and results, chest X-ray (CXR) findings, anticoagulation status before imaging, and CTPA outcomes reported by radiologists. Where the Wells score had not been documented, it was reconstructed retrospectively based on clinical notes to classify patients as either “PE likely” or “PE unlikely.” D-dimer values were categorized according to local reporting thresholds (<700 ng/mL, 700 to <1000 ng/mL, and ≥1000 ng/mL). CXR reports were reviewed to identify the presence of alternative diagnoses.

The primary outcome was diagnostic yield, defined as the proportion of CTPAs positive for acute PE. Secondary outcomes included documentation rates of the Wells score and D-dimer, the proportion of patients with alternative diagnoses on CXR, and anticoagulation patterns prior to imaging. Data were analyzed descriptively using Microsoft Excel, with categorical variables presented as frequencies and percentages. No statistical testing was undertaken, given the audit design and sample size.

Following review of the first-cycle findings, two interventions were implemented: the introduction of mandatory Wells score and D-dimer fields in the electronic CTPA request form on Cerner, and a presentation at the hospital Clinical Governance Meeting to improve awareness of appropriate CTPA requesting. The second cycle was subsequently performed to evaluate the impact of these measures on documentation quality and diagnostic performance.

## Results

Tables 1-4 show diagnostic yield, Wells score documentation, D-dimer testing, and CXR findings.

**Table 1:** Diagnostic yield CTPA, CT pulmonary angiography; PE, pulmonary embolism

Metric	Cycle 1 (October 2024)	Cycle 2 (September 2025)
Total CTPAs performed	58	96
Positive for PE	11	16
Diagnostic yield (%)	19	16.7

**Table 2:** Pretest probability PE, pulmonary embolism

Metric	Cycle 1	Cycle 2
Wells score documented	4/58 (6.9%)	84/96 (87.5%)
“PE likely” (Wells >4, includes documented and retrospectively calculated scores)	37 patients	61 patients
PE positive in “PE likely”	9/37 (24.3%)	14/61 (23%)
“PE unlikely” (Wells ≤4, includes documented and retrospectively calculated scores)	21 patients	23 patients
PE positive in “PE unlikely”	2/21 (9.5%)	2/23 (8.7%)

**Table 3:** Documentation of D-dimer PE, pulmonary embolism

Metric	Cycle 1	Cycle 2
D-dimer performed	45/58 (77.6%)	86/96 (89.6%)
Elevated D-dimer in PE-positive patients	5/5 (100%)	10/12 (83.3%)
PE-positive patients without D-dimer	0	4

**Table 4:** CXR findings CXR, chest X-ray

Metric	Cycle 1	Cycle 2
Alternative diagnosis on CXR	28/58 (48.2%)	25/96 (26%)

Cycle 1 (October 2024)

A total of 58 patients underwent CTPA for suspected PE during the first audit cycle. Eleven examinations were positive for PE, giving a diagnostic yield of 19%, which falls within the 15-35% range recommended by the RCR.

Documentation of the Wells score was very low. Only four of 58 patients had a recorded Wells score. Retrospective calculation classified 37 patients (63.8%) as “PE likely” (score >4) and 21 patients (36.2%) as “PE unlikely” (score ≤4). PE was confirmed in nine of 37 patients in the “PE likely” group (24.3%) and in two of 21 patients in the “PE unlikely” group (9.5%).

D-dimer testing was recorded in 45 patients (77.6%). All results were elevated according to local laboratory thresholds. No patients with a D-dimer level below 700 ng/mL had PE on CTPA. Higher D-dimer bands showed modestly increasing positivity rates, although the number of cases in each band was small.

CXR findings were reviewed for all patients. Alternative diagnoses were present in 28 patients (48.2%), most commonly pneumonia, chronic obstructive pulmonary disease exacerbation, pulmonary edema, or lobar collapse. Most patients with confirmed PE had normal CXR findings.

Cycle 2 (September 2025)

Ninety-six patients underwent CTPA for suspected PE in the second cycle. Sixteen examinations were positive, giving a diagnostic yield of 16.7%, similar to the first cycle and remaining within the recommended RCR range.

Wells score documentation improved substantially compared with the first cycle. Eighty-four of 96 patients (87.5%) had a documented Wells score. Among these, 61 patients (63.5%) were categorized as “PE likely,” with 14 confirmed PEs (23%), and 23 patients (24%) were categorized as “PE unlikely,” with two confirmed PEs (8.7%). This pattern of positivity was consistent with that observed in Cycle 1.

D-dimer testing was documented in 86 patients (89.6%), reflecting a marked improvement in recording. Among the 16 patients with confirmed PE, 12 had D-dimer testing available; 10 of these (83.3%) had elevated results. Four PE-positive patients did not have a recorded D-dimer.

Alternative diagnoses on CXR were identified in 25 of 96 patients (26%), a lower proportion than in Cycle 1. As in the first cycle, most patients with confirmed PE had normal CXRs, and many patients with abnormal CXRs did not have PE, reinforcing the importance of reviewing chest radiographs early in the assessment process.

Summary of change between cycles

Between the two audit cycles, documentation of the Wells score increased from four of 58 requests (6.8%) to 84 of 96 (87.5%), and D-dimer documentation increased from 45 of 58 cases (77.6%) to 86 of 96 cases (89.6%). Diagnostic yield remained stable at approximately 17-19%. While documentation improved markedly, overall patterns of PE positivity by Wells score category and alternative diagnoses on CXR were broadly similar across cycles.

## Discussion

This two-cycle audit evaluated adherence to national diagnostic guidance for suspected PE and assessed whether documentation quality and pathway compliance improved after a targeted local intervention. Across both audit cycles, the diagnostic yield for CTPA remained within the RCR’s expected range of 15-35%. However, substantial improvements in documentation of the Wells score and D-dimer were observed between October 2024 and September 2025, reflecting significant progress in adherence to the recommended diagnostic pathway.

In Cycle 1, Wells score documentation was notably poor, recorded in four cases out of 58 (6.9%). This is consistent with findings from other UK centers, where pretest probability assessment is frequently underdocumented despite being a core component of national guidance [9]. Retrospective reconstruction of the Wells score in Cycle 1 demonstrated a clear gradient in PE positivity between “PE likely” and “PE unlikely” categories, supporting its clinical utility and highlighting the importance of recording it prospectively. The marked improvement in Cycle 2, with 84 of 96 patients (87.5%) having a documented Wells score, indicates that the introduction of mandatory fields within the CTPA request form in Cerner had a sustained and meaningful effect on clinician behavior.

D-dimer documentation also improved substantially, rising from 45 of 58 cases (77.6%) in Cycle 1 to 86 of 96 (89.6%) in Cycle 2. In both cycles, D-dimer results were elevated in the majority of patients tested, but the yield remained relatively low in “PE unlikely” groups, reinforcing the need for appropriate interpretation of D-dimer within the context of pretest probability. The stronger documentation in Cycle 2 suggests that clinicians were more consistently applying the diagnostic pathway, even if overall imaging thresholds did not change.

Despite improved documentation, the diagnostic yield did not increase in Cycle 2. This stability suggests that although clinicians were recording Wells scores and D-dimer results more consistently, the decision to proceed with CTPA was not markedly altered. This may reflect persistent caution among clinicians when assessing patients with undifferentiated chest pain or dyspnea, the influence of medicolegal concerns, or the limitations of retrospective documentation in altering established clinical thresholds. Nonetheless, improved documentation strengthens the transparency of decision-making and ensures better auditability of practice.

CXR findings differed between the two cycles, with alternative diagnoses identified in 28 cases (48%) in Cycle 1 compared with 25 cases (26%) in Cycle 2. Although this difference may reflect variation in patient presentations over time, it also highlights the importance of reviewing CXRs prior to requesting advanced imaging. In both cycles, most patients with confirmed PE had normal CXRs, in keeping with established evidence [10], whereas many patients with abnormal CXRs ultimately did not have PE. This underscores the value of CXR as a triage tool and the need for clinicians to consider alternative diagnoses early in the assessment pathway [11].

This audit has several strengths. It included all CTPAs performed during the audit periods, eliminating sampling bias, and used consistent methodology across cycles, allowing meaningful comparison over time. The introduction of a system-level intervention between cycles strengthens the causal link between the changes implemented and the improvement observed. The improvement in documentation demonstrates the effectiveness of combining electronic prompts with educational reinforcement.

However, there are limitations. Retrospective reconstruction of Wells scores in Cycle 1 relied on the accuracy and completeness of clinical documentation, which may have introduced misclassification. Cycle 2 did not include detailed stratification of D-dimer bands or a full breakdown of anticoagulation status, limiting direct comparisons in these areas. Furthermore, although documentation improved significantly, this did not translate into an increase in diagnostic yield, suggesting that additional factors influence clinician decision-making when requesting CTPA. The extent to which the findings of this audit are generalizable to other institutions also remains uncertain.

These findings emphasize the importance of integrating decision-support elements into electronic request systems to align clinical practice with national guidelines. The improvement observed in Cycle 2 indicates that mandatory fields can act as effective safeguards to ensure key diagnostic steps are followed, in keeping with established literature [12]. Continued reinforcement through education, incorporation into departmental induction, and regular reauditing will be essential to maintain these gains. Future audits could explore whether improved documentation ultimately translates into reduced unnecessary imaging, shorter patient waiting times, or improved patient outcomes.

## Conclusions

This two-cycle audit demonstrated substantial improvements in adherence to national diagnostic guidance for suspected PE following the introduction of mandatory Wells score and D-dimer fields in the CTPA request form and the delivery of targeted educational initiatives. Documentation of both measures increased markedly between October 2024 and September 2025, indicating a sustained positive shift in clinician behavior and greater alignment with recommended diagnostic pathways. Although the diagnostic yield of CTPA remained stable across cycles, improved documentation enhances the transparency and auditability of decision-making and provides a stronger framework for ensuring appropriate imaging use. Continued monitoring, ongoing education, and periodic reaudits will be important to maintain these gains and further optimize CTPA utilization within the hospital.
